# Walking the tight wire between cell adhesion and WNT signalling: a balancing act for β-catenin

**DOI:** 10.1098/rsob.200267

**Published:** 2020-12-09

**Authors:** Tanne van der Wal, Renée van Amerongen

**Affiliations:** Developmental, Stem Cell and Cancer Biology, Swammerdam Institute for Life Sciences, University of Amsterdam, Science Park 904, 1098 XH Amsterdam, The Netherlands

**Keywords:** cell adhesion, WNT signal transduction, β-catenin, e-cadherin, interplay, epithelial–mesenchymal transition

## Abstract

CTNNB1 (catenin β-1, also known as β-catenin) plays a dual role in the cell. It is the key effector of WNT/CTNNB1 signalling, acting as a transcriptional co-activator of TCF/LEF target genes. It is also crucial for cell adhesion and a critical component of cadherin-based adherens junctions. Two functional pools of CTNNB1, a transcriptionally active and an adhesive pool, can therefore be distinguished. Whether cells merely balance the distribution of available CTNNB1 between these functional pools or whether interplay occurs between them has long been studied and debated. While interplay has been indicated upon artificial modulation of cadherin expression levels and during epithelial–mesenchymal transition, it is unclear to what extent CTNNB1 exchange occurs under physiological conditions and in response to WNT stimulation. Here, we review the available evidence for both of these models, discuss how CTNNB1 binding to its many interaction partners is controlled and propose avenues for future studies.

## 1. Introduction

CTNNB1 (catenin β-1, also known as β-catenin) plays a dual role in the cell. It is a core component of the WNT/CTNNB1 pathway, which is crucial for tissue morphogenesis and maintenance throughout the lifespan of all multicellular animals. In this capacity, it functions as a transcriptional co-activator of TCF/LEF target genes. It also has a key function at cell–cell junctions, where it is required to anchor cadherins to the cytoskeleton, forming the essence of cell adhesion. This duality in function poses an interesting conundrum that has intrigued scientists for decades. All complex animals need to maintain adhesive, intact tissues, while at the same time they must tightly regulate tissue-specific gene expression programmes; how do cells employ the same protein for both tasks?

Historically, the dual functions of CTNNB1 have mostly been studied from either a WNT or cell adhesion perspective [1–3], and it is clear that two functionally distinct pools exist. However, in both fields, it remains an outstanding question how cells balance the distribution of CTNNB1 across these functional pools and if interaction may occur between them (as previously reviewed by Heuberger & Birchmeier [4]; Daugherty & Gottardi [5]; McEwen *et al*. [6]). For instance, during development, transcriptionally active CTNNB1 is crucial for patterning to induce correct cell identity and tissue morphology, but at the same time CTNNB1 is needed at the cell–cell junctions to link cells and maintain tissue integrity while these morphological changes are actually occurring. In adult tissues, this balance is just as important to preserve tissue homeostasis, and fluctuations in either direction can cause cells to change their transcriptional programme or alter their adhesion, which are all common processes in diseases such as cancer or during tissue injury and repair.

Here, we summarize the current state of knowledge on how cells dynamically distribute CTNNB1 between the adhesion and transcriptional pool, discuss limitations of current experimental approaches and suggest future directions for research.

## 2. CTNNB1: a molecular multiplayer

The many cellular activities of CTNNB1 have previously been reviewed in great detail [7]. For the purpose of this review, we will refer to the two functionally distinct pools as the ‘adhesion’ and the ‘transcriptional’ pool of CTNNB1. Additionally, it is important to realize that CTNNB1 not only has two different functions, but is also present at different locations within the cell. Importantly, its function and location do not necessarily overlap.

### 2.1. Function of CTNNB1

**Figure 1.**
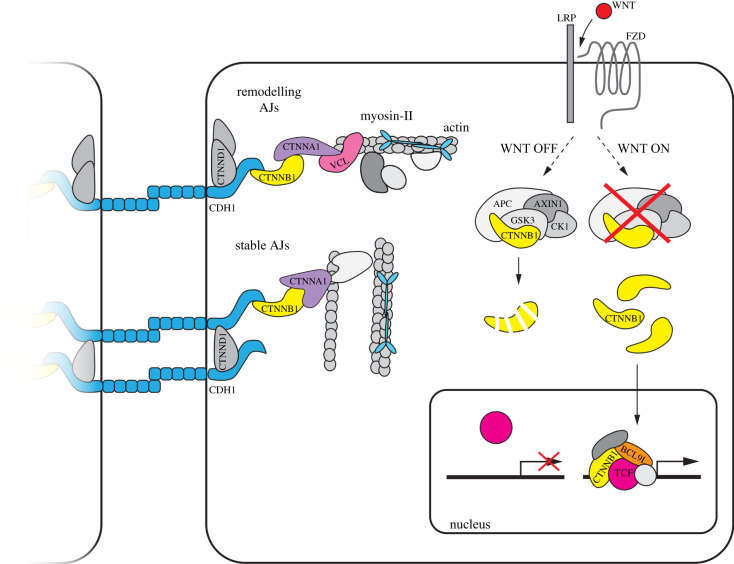
Schematic of CTNNB1 in cell adhesion and WNT/CTNNB1 signalling. Most schematic depictions of WNT/CTNNB1 signalling do not display the large CTNNB1 fraction at cell–cell junctions, and vice versa. This figure visualizes both the structural role of CTNNB1 in cell adhesion and the transcriptional role of CTNNB1 in WNT/CTNNB1 signalling. AJs can have different conformations, including a stable and remodelling state. AJs are formed by cadherins that bind their counterparts on adjacent cells via their extracellular domains and are attached to the cytoskeleton through their intracellular tails via CTNNB1 and CTNNA1. In remodelling AJs, the actin/myosin bundles run perpendicular to the membrane, and VCL is present. In stable AJs, actin/myosin bundles run parallel to the membrane. CTNNB1 is also a critical component of the WNT/CTNNB1 pathway. In the presence of WNT signals, it activates the transcription of WNT target genes in the nucleus together with TCF as part of the enhanceosome. In the absence of WNT, CTNNB1 is sequestered in the cytoplasm by the destruction complex, consisting of APC, AXIN, CK1 and GSK3, where it gets phosphorylated and subsequently degraded by the proteasome.

CTNNB1 is the main downstream effector of the WNT/CTNNB1 pathway (figure 1). It functions as a co-activator for TCF/LEF transcription factors in the nucleus to modulate the expression of WNT target genes. WNT/CTNNB1 signalling is a highly conserved signal transduction pathway that is crucial for embryonic development and adult tissue maintenance in all multicellular animals. The field has gotten too complex to cover comprehensively. Specific aspects, including the evolutionary and developmental complexity of the WNT/CTNNB1 pathway and its pathological implications, have been discussed elsewhere [8–12]. Here, we will focus on the latest model of the core signalling mechanism, as also concisely summarized by Gammons & Bienz [13].

In the absence of WNT signals, CTNNB1 is sequestered in the cytoplasm by a multiprotein complex consisting of the scaffolding proteins APC and AXIN1, and serine/threonine kinases CK1 and GSK3 [14–18]. Once bound, CTNNB1 is sequentially phosphorylated on residues S45 (priming phosphorylation by CK1), T41, S37 and S33 (subsequent phosphorylation by GSK3), leading to its ubiquitination and proteasomal destruction [19–21]. This molecular machinery helps maintain low levels of CTNNB1 in the cytoplasm and nucleus in the absence of WNT stimulation.

Historically referred to as the CTNNB1 destruction complex and recently also called the ‘AXIN degradosome’ [13], the CTNNB1 destruction machinery can form larger biomolecular condensates in the cytoplasm [22,23]. Driven by the structural properties of both AXIN and APC, which can not only self-polymerize but also undergo multivalent interactions with multiple different binding partners, local concentration of destruction complex components can occur as a result of liquid–liquid-phase separation [13,24].

Binding of WNT to FZD and LRP receptors at the plasma membrane results in the formation of the so-called WNT signalosome [25]. This involves the clustering of FZD/LRP receptor complexes, which is at least partially driven by the self-polymerization of membrane-associated DVL proteins at the cytoplasmic interface. Of note, DVL and AXIN1 can form heterologous interactions via their respective DIX and DAX domains. As a result, DVL not only inhibits AXIN1 self-assembly but also competes with APC for binding to AXIN1 [26,27]. Moreover, the cytoplasmic tail of the LRP co-receptor, once phosphorylated, forms binding sites for AXIN1 and GSK3 [25]. This series of events, centring around the recruitment of AXIN1 to the plasma membrane [28], causes inactivation of the destruction complex and leads to an increase in nuclear and cytoplasmic CTNNB1 levels [25,29–33]. Importantly, it remains to be determined whether the entire destruction complex is recruited to the plasma membrane, whether it dissolves en route, or whether only the formation of new destruction complexes is prevented by the recruitment of AXIN and GSK3. This inhibition of the destruction complex is a crucial step in WNT/CTNNB1 signalling. Other nodes of regulation, including active translocation to and from the nucleus and active nuclear retention, are important as well. Together, they ensure that CTNNB1 levels specifically increase in the nucleus in response to WNT stimulation [34].

In the nucleus, CTNNB1 and TCF/LEF associate with other proteins, such as BCL9/BCL9 L and PYGO1/2, to form a larger transcriptional regulatory complex [35–40]. Also termed the ‘WNT enhanceosome’ [41], it is responsible for the tissue-specific and context-dependent activation of WNT target gene programmes [38,42], although it should be noted that the transcriptional co-activator activities of CTNNB1 extend far beyond TCF/LEF alone (as excellently reviewed by Söderholm & Cantù [43]).

In all multicellular animals, cell–cell junctions maintain the structural integrity and morphology of tissues, which are crucial for their proper functioning [44,45]. CTNNB1 is a central component of adherens junctions (AJs) (figure 1), a specific class of cell–cell junctions and the main providers of tissue stability [45,46]. AJs link cells by forming extracellular bonds with neighbouring cells and anchoring these bonds to the cytoskeleton. Intercellularly, AJs are connected via the extracellular domain of cadherin transmembrane proteins, with CDH1 (cadherin-1, also known as E-cadherin) and CDH2 (cadherin-2, also known as N-cadherin) as the best-known examples [47,48]. CTNNB1 binds to the C-terminal, cytoplasmic domain of cadherins and links them to CTNNA1 (catenin α-1), which in turn anchors the junctions to the cytoskeleton [49,50]. CTNNB1 thus performs a critical role in the anchoring of the AJs, without which the junctions would lose their tension and structure. Another armadillo protein, JUP (junction plakoglobin, also called catenin γ), can also bind the C-terminal cytoplasmic domain of cadherins and perform a similar role as CTNNB1 [51]. Although it can compensate for the loss of CTNNB1 under some circumstances, JUP is typically found in desmosomes, rather than AJs [52,53].

Anchoring to the cytoskeleton occurs either through direct binding of CTNNA1 to F-actin or via other actin-binding proteins that interact with CTNNA1, such as VCL (Vinculin) [54,55]. Tension is needed for anchoring of either CTNNA1 or VCL to F-actin to occur: binding follows a so-called two-state catch bond model, in which an interaction only forms under intermediate tension between the interacting partners [56–59]. Depending on the developmental and morphological state of the cell, AJs can have different conformations with alternative tensional characteristics and contain additional proteins that help stabilize or remodel the junctions. For example, VCL is mainly observed in remodelling AJs and in the zonula adherens found in mature epithelial cells, which are under higher tension than other AJ types [60]. CTNND1 (catenin δ-1, also called p120-catenin), a third armadillo protein involved in cell adhesion, is an example of a stabilizing protein that binds the juxtamembrane region of the cytoplasmic tails of cadherins to further strengthen the junctions [61–63].

The combination of both transcriptional and adhesive properties in one single CTNNB1 protein is a feature that has been conserved across evolution in animals as distantly related as mammals and sponges [64]. Whether the transcriptional or adhesive functions arose first, or perhaps simultaneously, remains unknown [65–67]. The two functions can be separated, as evidenced by the existence of multiple CTNNB1 homologues in *Caenorhabditis elegans*, some of which are exclusively involved in transcription or adhesion [68–70]. On a final note, a CTNNB1/CTNNA1 module regulates polarization of a simple epithelium in the slime mold *Dictyostelium discoideum* at a transient, multicellular life stage [71]. This role for CTNNB1 and CTNNA1 in cell polarization may thus pre-date both WNT/CTNNB1 signalling and cadherin-mediated cell–cell junctions [71].

### 2.2. Location of CTNNB1

Although CTNNB1 clearly fulfils its two functions in cell adhesion and WNT signalling at distinct locations within the cell, the location of CTNNB1 does not necessarily say anything about its functional activity. For instance, while the transcriptionally active pool of CTNNB1 by definition must be present in the nucleus, not all nuclear CTNNB1 has to be transcriptionally active. Similarly, while it is safe to assume that the functional adhesion pool of CTNNB1 will be located at the cell membrane, not all CTNNB1 present at the membrane is automatically part of an AJ.

In CDH1-null Kep1 cells, an increase in dephosphorylated CTNNB1 levels at the membrane upon WNT stimulation could be observed [72]. Since membrane-localized CTNNB1 co-localized with APC, AXIN and LRP6, this could reflect the WNT-mediated recruitment of destruction complex components to the FZD/LRP receptor complex, although other mechanisms may be at play. Around the same time, the presence of AXIN and phosphorylated CTNNB1 at the membrane was shown to occur in SW480 cells with both low and high expression of CDH1 [73]. While the presence of destruction complex components at the cell membrane can be explained by their role in signalosome formation, if and how CTNNB1 turnover is still regulated at the membrane by these components remains unknown. Technically speaking, CTNNB1 itself could be part of the WNT signalosome at least transiently.

**Figure 2.**
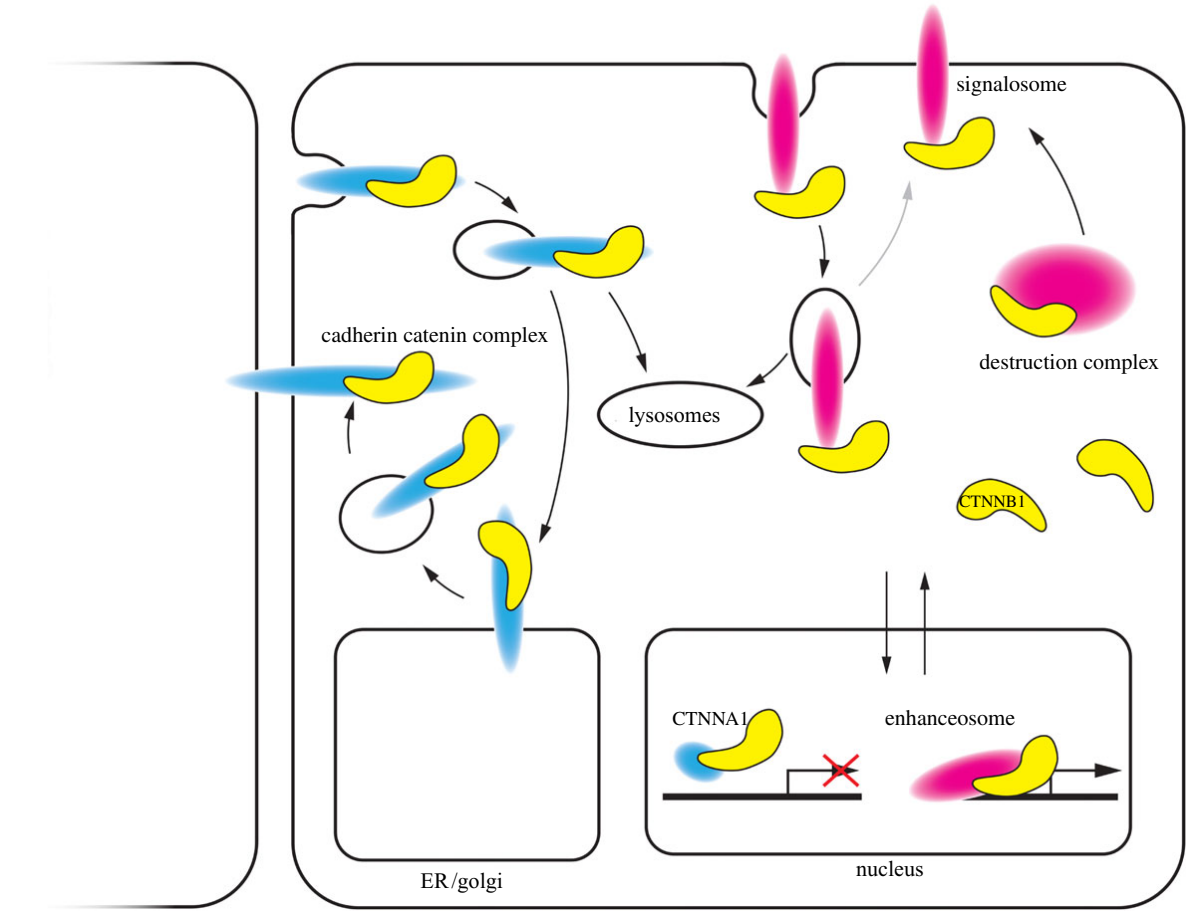
Schematic visualization of the localization of CTNNB1 functional pools. CTNNB1 (yellow) functional pools cannot be distinguished based on their localization. The adhesion pool of CTNNB1 (blue) is present at the membrane, but also undergoes anterograde and retrograde trafficking. Furthermore, CTNNA1/CTNNB1 dimers have been shown to be present in the nucleus, but they are not transcriptionally active. The transcriptional pool of CTNNB1 (pink) is present in the nucleus to activate transcription. It is also present in the cytoplasm as free CTNNB1, and as bound CTNNB1 sequestered by the destruction complex. Finally, the transcriptional pool of CTNNB1 may be present at the membrane, possibly via interaction with the WNT signalosome.

Using a stable HEK293 cell line with the low-level overexpression of fluorescently tagged CTNNB1, Kafri *et al*. [74] showed that CTNNB1 levels at the membrane indeed increase upon WNT stimulation, although to a lesser extent than the concomitant increase in the cytoplasm and nucleus, which is also known to be modest [34]. CTNNB1 dynamics at the membrane were found to be stable (as typically observed for AJs) and unchanged in the absence and presence of WNT stimuli [74]. However, as further elaborated below, CDH1 is degraded unless it is bound by CTNNB1 [75,76], so there should be very few unsaturated CDH1-binding sites at the cell membrane to accommodate an absolute increase in CTNNB1 levels, again supporting the potential existence of a membranal CTNNB1 pool unrelated to cell adhesion. To summarize, the presence of CTNNB1 in the signalosome at the membrane would induce a second pool of CTNNB1 at the membrane with unknown dynamics and stability (figure 2).

Conversely, not all cytoplasmic CTNNB1 is active in the WNT signalling pathway. For example, CTNNB1 and CDH1 associate immediately upon synthesis. This early interaction between CTNNB1 and the cytoplasmic C-terminal tail of CDH1 appears to stabilize CDH1, which rapidly undergoes proteolysis in the absence of CTNNB1 binding [75,76]. CTNNA1 was initially reported to only become associated with the cadherin complex upon arrival of CDH1 at the cell membrane [77,78]. However, it was later shown that both CTNNA1 and CTNNB1 associate with CDH2 after phosphorylation of the C-terminal tail, but prior to proteolytic processing of the cadherin precursor protein [79]. Because CTNNA1 and CTNNB1 can also be detected as dimers in the cytosol [79], they might be loaded onto the cadherin complex simultaneously. Later work demonstrated that the vast majority of immature CDH1 and CDH2 proteins is associated with CTNNA1 and CTNNB1 [80], presumably already in the endoplasmic reticulum, and these complexes thus undergo anterograde trafficking to the plasma membrane together. Moreover, both AJs, which undergo recycling [81] and components of the destruction complex or signalosome, also undergo endocytosis and retrograde trafficking [82]. These events introduce a dynamic, vesicular cytoplasmic component of CTNNB1 (figure 2). Monomeric, N-terminally unphosphorylated—and presumably signalling competent—CTNNB1 represents a free cytoplasmic pool, while destruction complex-bound CTNNB1 is contained in cytoplasmic biocondensates (figure 2). Without explicit characterization of the complex and phosphorylation state that CTNNB1 resides in, it can therefore be difficult—if not impossible—to define a functional pool of CTNNB1 based on its localization, both with microscopy-based and biochemical approaches.

### 2.3. The trouble with CTNNB1

The duality in function of CTNNB1 has been a topic of debate for years. Researchers have attempted to study the balanced distribution or possible interaction between the functional CTNNB1 pools using a variety of techniques and model systems, all with their own advantages and limitations. The ill-corresponding relationship between the function and location of CTNNB1 poses a great limitation in the interpretation of these studies, as localization by itself is not sufficient to determine the functional identity of a particular protein pool. To date, there is no consensus regarding if, how and when the two pools of CTNNB1 interact—or, if they do not interact, how a cell separates the functions of CTNNB1.

## 3. Experimental evidence for separation of the CTNNB1 pools

If two totally different functions are carried out by the same protein and those two functional pools of CTNNB1 do not interact, the question of how cells maintain a proper balance in the distribution of CTNNB1 naturally arises. One obvious mechanism would be via binding partners, which CTNNB1, as an armadillo protein, has many of. Below, we have listed several known CTNNB1 interactors and the post-translational modifications regulating these interactions in addition to post-translational modification sites on CTNNB1, the function of many of which remains unknown (table 1).

**Table 1.** Comprehensive overview of regions and critical residues that determine CTNNB1 binding and localization. The CTNNB1 protein is composed of 781 residues (first column) divided into different structural regions (second column). The central core of 12 armadillo repeats is flanked by a more flexible N-terminal domain (NTD) and a C-terminal α-helix (Helix C) and a flexible C-terminal domain (CTD). Although the binding interface for its many interaction partners spans a larger portion of the protein and thus overlaps, smaller critical regions (third column) and even critical residues (fourth column) have been mapped that are responsible for most of the binding affinity of a specific interaction. Different post-translational modifications (PTMs) of specific residues can alter the distribution of CTNNB1 across different subcellular and/or functional pools (columns five through eight). The responsible modifying enzymes have been identified for many of these events. This table was compiled using information from the human (https://www.uniprot.org/uniprot/P35222) and mouse (https://www.uniprot.org/uniprot/Q02248) UniProt pages (accessed 20 June 2020) and indicated references from the literature (last column), in combination with the different CTNNB1 protein crystallography studies referred to in the main text.

CTNNB1			critical residue					reference
			mutagenesis	PTM				
residue	region	binding partner (critical region)	mutation (effect)	membrane	cytoplasm	nucleus	modifier (effect)	
1–150	NTD	VCL (2–23)	M8P: VCL lost (less AJs)					[83]
			???		K19^Ub^		SCF ubiquitin ligase	[84]
			???	S23^O−Glc−Nac^	S23^P^	S23		[85]
			S29A (+T102A +T112Q)	S29^P^	S29		CK2 (increases CTNNA1 binding)	[86]
			D32G/G34E		DS29^P^GXXS37^P^		BTRCP (degradation)	[87,88]
			S33Y (more CTNNB1)		S33^P^		GSK3 (degradation)	[20,21]
			S37A (more CTNNB1)		S37^P^		GSK3 (degradation)	[20,21]
			T41A (more CTNNB1)		T41^P^		GSK3 (degradation)	[20,21]
			S45F (more CTNNB1)		S45^P^		CKI (degradation)	[20,21]
			???			K49^Ac^	CBP (more transcription)	[89]
			???	Y64^P^		Y64^P^	PTK6 (?)	[90]
			???		Y86 (retention)	Y86^P^	BCR-ABL (more transcription)	[91,92]
			S29A (+T102A +T112Q)	T102^P^		T102	CK2 (increases CTNNA1 binding)	[86]
			S29A (+T102A +T112Q)	T112^P^		T112	CK2, PKD1 (increases CTNNA1 binding)	[86]
		CTNNA1 (120–151)	T112R/T120I	T120^P^		T120	PKD1 (CTNNA1 binding)	[93,94]
			Y142E, Y142A	Y142		Y142^P^	FYN/FER/cMET/PTK6 (CTNNA1 binding lost)	[95,96]
151–191	ARM 1	BCL9 (156–178)	L156A+L159A (BCL9 binding lost) D164A+deltaC (adhesion preserved, signalling lost)					[97,98]
						S191^P^	JNK2 (more nuclear) CDK5 (???)	[99,100]
193–234	ARM 2							
235–276	ARM 3	AXIN1/2	F253A: AXIN2 lost H260A: AXIN1/2 lost		S246^P^		CDK5 (less APC binding)	[100]
277–318	ARM 4	AXIN1/2	K292A: AXIN1/2 lost K312E: TCF7L2 lost					
319–360	ARM 5	APC	K345A: APC lost K345R: TCF7L2 reduced			Y333^P^ K345^Ac^	Y333 (PTK6, SRC: more nuclear) ???	[90,101] [102]
361–389	ARM 6	APC	W383: APC lost					
			R386A: APC lost					
			T393D (P-mimic, more stable)		T393^P^		CK2 (less AXIN binding)	[99]
			T393A (mutant)					
400–441	ARM 7		N426A: LEF1 lost K435A: LEF1/TCF7L2 lost					
442–484	ARM 8	TCF/LEF	H470A: TCF7L2/LEF1 lost					
489–530	ARM 9		K508A: LEF1 lost	Y489	Y489^P^	Y489^P^	ABL (less CDH2 binding)	[103]
531–571	ARM 10		stabilizes CTNNB1			S552^P^	AKT (more nuclear)	[104,105]
					T556^P^		NEK2 (stabilization at the centrosome)	[106]
594–636	ARM 11			C619^S−NO^		S605^P^	??? (reduced adhesion)	[99] [107]
637–666	ARM 12		Y654E: enhanced transcription Y654F: TBP binding lost	Y654		Y654^P^	CSK, SRC, EGFR	[91] [92,108]
				K666^Ub^			SIAH (degradation)	[109]
667–683	Helix C	transcriptional co-activators	D164A+deltaC (adhesion preserved, signalling lost)			S675^P^	PKA (more nuclear)	[105,110,111]
				Y670 K671^Ub^		Y670^P^	??? (cMET binding) SIAH (degradation)	[108] [109]
684–781	CTD	transcriptional co-activators SCRIB (772–781)	D164A+deltaC adhesion preserved, signalling lost)					

### 3.1. Interacting partners of CTNNB1

CTNNB1 was the first identified armadillo protein [112], a large family of evolutionary conserved proteins with diverse functions (as excellently reviewed by Fagotto [113]). All armadillo proteins, including CTNNB1 and its close homologues JUP and CTNND1, are characterized by the presence of tandem-repeated ARM motifs, amino acid sequences of approximately 42 residues in length [112,114,115]. CTNNB1 contains a series of 12 arm motifs, each consisting of α-helices. Together, these ARM repeats form a superhelical structure [115,116]. This conformation is characteristic for armadillo proteins and presumably aids the binding of multiple interacting proteins, as typically observed for armadillo family members [116–118].

**Figure 3.**
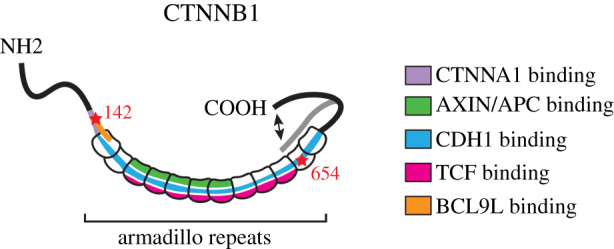
Schematic visualization of CTNNB1 and its key binding sites for interaction partners. CTNNB1 contains 12 armadillo repeats, which among others contain binding sites for cadherins, TCF, AXIN, APC and BCL9 L. These binding sites overlap, creating competition for binding. CTNNA1- and BCL9 L-binding sites at the NH2 terminus also overlap. The flexible C-terminal domain has been proposed to be able to change conformation, thus blocking the cadherin-binding site.

Indeed, CTNNB1 has a diverse range of interacting partners, some of which compete for binding due to overlapping binding domains (figure 3 and table 1). As for its function in cell adhesion, CTNNB1 contains a binding domain for both the cytoplasmic domains of cadherins and CTNNA1, the two main components of AJs [119,120]. To perform its transcriptional role in WNT signalling, CTNNB1 contains a TCF-/LEF-binding domain [35,38]. This binding domain overlaps with that for CDH1, meaning that CTNNB1 can bind either CDH1 or TCF and can therefore only fulfil one function at the time [121]. In addition, CTNNB1 can also bind destruction complex components such as AXIN and APC and a range of transcriptional co-activators and WNT enhanceosome components, such as BCL9/BCL9 L [98,119,122,123]. Over the years, the molecular details of these interactions have been resolved in great detail by a combination of protein mutagenesis, *in vitro* binding studies and crystallography approaches [76,97,124–126].

### 3.2. Functional biochemical studies

While multiple proteins compete for binding to overlapping domains on CTNNB1, different hot spots can be mapped that constitute high-affinity interaction sites with a critical role for specific amino acids on CTNNB1. Some of these residues have been shown to be subject to post-translational modification, which can have a major impact on binding affinity. For instance, SRC-mediated tyrosine phosphorylation of CTNNB1 residue Y654 affects binding of CTNNB1 to CDH1, the predominant cadherin molecule in epithelial tissues, and reduces this interaction approximately sixfold [92]. A different residue, Y489, can be phosphorylated by ABL, resulting in reduced affinity for CDH2, the predominant cadherin in neuroectodermal cells [103]. In addition, Y142 can be phosphorylated by multiple kinases, leading to the release of CTNNB1 from CTNNA1 [96]. Residues Y654 and Y142 therefore appear critical for CTNNB1 to fulfil its function in cell adhesion [127].

Because the CTNNB1-binding domains for cadherins and TCF partially overlap, competition for these sites is created [121]. In the absence of additional post-translational modifications or regulatory partners for CTNNB1, this would imply that the sole availability of and affinity for CTNNB1-binding partners would regulate its distribution. Indeed, this was originally hypothesized when it was observed that altering cadherin levels affected CTNNB1 distribution [128–133]. As CTNNB1 and CDH1 can bind immediately upon synthesis (see §2.2), increasing CDH1 production could thus be a very direct way of ensuring that most of the newly synthesized CTNNB1 traffics to the membrane, rather than to other locations in the cell.

To switch between its transcriptional and adhesive functions, CTNNB1 has to preferentially bind either TCF/LEF or cadherins [95,134]. Based on *in vitro* pull-down assays with deletion mutants, Gottardi & Gumbiner [134] proposed an elegant model, in which a conformational change of the CTNNB1 C-terminus upon WNT pathway activation obscures the cadherin-binding domain but not the TCF-binding domain, ensuring preferential binding to TCF under these circumstances. Indeed, in the presence of WNT stimulation, cells preferentially bound soluble TCF domains over soluble cadherin cytoplasmic domains [134]. The precise molecular mechanism responsible for this presumed conformational change remains to be determined, but it probably requires one or more of the aforementioned post-translational modifications (table 1).

Of note, an active role has been proposed for CTNNA1 in maintaining the separation between the adhesive and transcriptional pools of CTNNB1 independent from cadherin-binding. Monomeric CTNNB1 preferentially binds to TCF instead of CDH1, but can still bind to CTNNA1, and it was hypothesized that these CTNNB1/CTNNA1 dimers can travel to the nucleus, but cannot activate gene expression [134]. Indeed, CTNNB1 and CTNNA1 have both been detected in the nucleus and binding of CTNNA1 to CTNNB1 indeed blocks its transcriptional activity [95,135,136]. As such, CTNNA1 may thus be able to deliver a nuclear supply of CTNNB1 that is kept in an inactive form until its transcriptional activity is needed. Here, Y142 plays a critical role, since phosphorylation of this residue releases CTNNA1 from binding. This allows the transcriptional co-factor BCL9 L, which has an overlapping binding site on CTNNB1, to bind instead [95], even if phosphorylation of Y142 is not critical for the interaction with BCL9 L itself [97,137].

Altogether, many CTNNB1 residues can be post-translationally modified, not just by phosphorylation but also via ubiquitination, sumoylation, acetylation, glycosylation and nitrosylation (table 1). For many of these modifications, the exact role remains to be determined. Generally speaking, post-translational modifications are a rapid way to modify protein function by subtly altering binding affinities and they could thus allow cells to dynamically switch and appropriately balance the distribution of CTNNB1 across different pools, depending on the cellular context. It is likely that we have only begun to uncover the tip of the iceberg, let alone understand this layer of regulation. For instance, *S*-nitrosylation of residue C619 in CTNNB1 was shown to occur in endothelial cells in response to nitric oxide. This resulted in weakening of the endothelial AJs and increased permeability of the blood vessels [107].

How these different regulatory mechanisms are integrated requires further research. However, together they equip cells with sensitive and versatile mechanisms to fine-tune the distribution of CTNNB1 across its different functional pools in different biological settings.

## 4. Experimental evidence for interplay of the CTNNB1 pools

Despite the clear need to functionally separate the adhesive and transcriptional pools of CTNNB1, evidence for functional interplay also exists. Most of this evidence comes from very specific situations and experimental conditions in which cell adhesion changes occur.

### 4.1. Overexpression and knockout studies

Interplay between the different pools of CTNNB1 was first mentioned in the late 1990s, following overexpression and knockouts of cadherins. This was a serendipitous discovery at the time, since these studies were actually aimed at identifying the mechanism responsible for the rapid turnover of cytoplasmic CTNNB1 in the absence of WNT pathway activation—a role which we now know to be performed by the destruction complex.

Overexpression of cadherins in *Xenopus* embryos was shown to inhibit CTNNB1 transcriptional activity, as judged by the resulting phenotypes. Developmental abnormalities were only observed when cadherins were overexpressed in embryonic areas that required active WNT/CTNNB1 signalling for normal development and could be rescued by injection of CTNNB1 [129,131]. Furthermore, overexpression of both CTNNB1 and cadherins resulted in increased localization of CTNNB1 at the cell membrane, as demonstrated by immunofluorescence staining [129]. Similar results were obtained in *Drosophila*, where overexpression of the full-length fly E-cadherin protein *shotgun* or soluble *shotgun* cytoplasmic domains leads to phenotypes similar to those in *wingless* loss-of-function mutants [133]. These results were confirmed in human CHO and SW480 cell lines by overexpressing soluble CDH1 and CDH2 cytoplasmic domains [132]. The latter study also confirmed that cadherin overexpression had an inhibitory effect on CTNNB1 transcriptional activity using a LEF-1 luciferase reporter as an experimental readout. In agreement with these findings, depletion of cadherins leads to increased levels of WNT signalling activity in *Drosophila* embryos and human embryonic stem cells [121,128]. However, this is not always the case as multiple studies have reported that CDH1 null cancer cell lines do not show constitutive WNT signalling activity [138–140]. It is seemingly counterintuitive that the loss of CDH1 does not automatically cause enhanced WNT/CTNNB1 signalling and this remains a conundrum. Sufficient regulatory mechanisms probably remain in place to prevent aberrant WNT signalling activation in these cell lines. It could also be that these cancer cell lines have adapted to the long-term, constitutive loss of CDH1. In a more complex context, such as the developing *Drosophila* embryo, changes in cadherin levels also occur in the presence of exogenous WNT signals, which may tip the balance towards WNT/CTNNB1 pathway activation. As such, cancer cell lines with mutations in CDH1 may not represent the subtle dynamic changes occurring *in vivo*.

Together, these studies showed that, when present in sufficiently high amounts or even in excess, cadherins can sequester CTNNB1 at the membrane, thereby inhibiting its transcriptional function in WNT signalling. While these discoveries were made under somewhat artificial conditions, they had important ramifications: although mechanisms to balance the distribution of CTNNB1 between its two functional pools exist, crosstalk might be possible between them. To what extent cell adhesion directly influences CTNNB1 transcriptional activity has thus been a question that has been asked for many decades [141]. It remains to be resolved all these years later. Whether more physiologically relevant modulations of cell adhesion affect the distribution of CTNNB1 in the transcriptional functional pool has mainly been studied in the context of epithelial–mesenchymal transition (EMT).

### 4.2. EMT studies

One obvious example in which the distribution of CTNNB1 across its functional pools may shift is during EMT. During this naturally occurring and reversible process, epithelial cells adopt a mesenchymal phenotype, altering their transcriptional programmes, cell shape and migratory behaviour (see box 1). EMT, and its reverse process MET, is required at multiple stages of normal embryonic development, but can also be pathogenic in disease, and is an important driver of the metastatic cascade.

Box 1. **Hallmarks of EMT.**

So-called cadherin switching has long and widely been considered a hallmark of the EMT process. From a molecular perspective, it is a common way for cells to separate and sort themselves during tissue morphogenesis [142]. The classical example is that in which the loss of *CDH1* (encoding E-cadherin) expression and gain of *CDH2* (encoding N-cadherin) expression coincides with the loss of cell–cell contacts, including adherens junctions, and the gain of mesenchymal characteristics, including migratory behaviour.

Downregulation of epithelial CDH1 occurs on both the transcriptional and the protein level (as reviewed by Lamouille *et al.* [143] and Yilmaz & Christofori [144]). Transcriptional inhibition is driven by EMT transcription factors, such as SNAI1, SNAI2 and TWIST1. While transcriptional inhibition ensures that no new junctions are formed, active removal of cadherin molecules from the cell surface ensures that existing junctions are broken down. This removal occurs through endocytosis, followed by lysosomal degradation of the cadherins, which normally undergo recycling [145,146].

An international consortium of researchers recently published guidelines to investigate and define the EMT process, and its many intermediate phenotypes, based on a combination of molecular markers and cellular properties [147]. Despite the complexity and context-dependent characteristics of the EMT programme, the consensus remains that epithelial cells weaken or lose their adherens junctions during the onset of EMT as a common feature, although it is still not entirely clear whether this is a cause or consequence of the process.

EMT model systems (see the main text for examples) allow direct and extreme modulation of cell adhesion conditions. This provides an attractive experimental setting to study the interplay between cell adhesion and WNT/CTNNB1 signalling—obviously, the downregulation of CDH1 directly reduces the number of CTNNB1-binding sites at the plasma membrane and thus a larger CTNNB1 pool could become available for signalling—as the molecular details and true extent of this interplay remain incompletely understood. Finally, some of the master regulatory EMT transcription factors have themselves been shown to be direct transcriptional targets of WNT/CTNNB1 signalling, suggesting that an EMT regulatory gene circuit could also contribute to crosstalk between the different functional pools of CTNNB1 in a more indirect manner [148–150].

The dynamic response of CTNNB1 to an EMT induction has been studied in several *in vitro* assays modelling EMT, such as hepatocyte growth factor (HGF)-induced EMT and wound scratch assays. MDCK cells are often used as an epithelial cell line in this context as they form multicellular sheets in culture. However, it should be kept in mind that in the context of WNT signalling, MDCK cells can be less responsive than some other commonly used cell lines such as HEK293 cells [151].

In MDCK cells, CTNNB1 levels increase in the cytoplasm and nucleus upon HGF-induced EMT and wound healing-induced sheet migration [151,152]. This increase was recently shown to be directly due to translocation of CTNNB1 from the membrane: using a photoconvertible fluorescent protein-tagged version of CTNNB1, a proportion of the membrane localized pool of CTNNB1 could be visualized and tracked to follow its dispersion in the cell upon HGF stimulation [152]. The problem, of course, is that this localization-based experiment does not directly demonstrate that it is the cadherin-associated pool of CTNNB1 that is released.

One would logically assume that an increase in cytoplasmic and nuclear CTNNB1 levels automatically corresponds to increased CTNNB1-mediated transcriptional activity. Indeed, both studies showed that upon CTNNB1 translocation, an increase in CTNNB1 transcriptional activity and target gene expression occurs [151,152]. However, when CDH1 was depleted, Howard *et al*. [151] found that the resulting cytoplasmic increase of CTNNB1 no longer leads to an increase in CTNNB1 transcriptional activity, suggesting that CDH1 might even somehow be required for WNT/CTNNB1 signalling activation. Moreover, phosphomimic CTNNB1 Y654E mutants, which have a lower affinity for CDH1 and therefore tend to localize to the cytoplasm, showed a lower transcriptional activity than wild-type CTNNB1 in both the absence and presence of HGF [151]. Thus, increased cytoplasmic levels of CTNNB1 after EMT do not automatically correspond to equal amounts of transcriptionally active CTNNB1. This again shows the importance of functional readouts, as localization alone does not define the function of CTNNB1.

As to the mechanism and regulatory nodes responsible for the dissociation of CTNNB1 from the membrane in these studies, some discrepancies remain. Upon HGF stimulation, Howard *et al*. [151] found endocytosis to be required for the joint release of CTNNB1 and CDH1 from the membrane. Following endocytosis, CTNNB1 and CDH1 dissociate and part ways to the perinuclear region and lysosomes, respectively. Supposedly, phosphorylation of both CDH1 and CTNNB1 aids their dissociation, and this involves the aforementioned SRC-mediated phosphorylation of Y654 on CTNNB1. Gayrard *et al*. [152] propose an alternative mechanism, in which release of tension exerted on CDH1 is required for CTNNB1 dissociation. Although SRC was found to be constitutively active after HGF stimulation, phosphorylation of the SRC-target residue Y654 was not [152]. Instead, SRC activation was found to lead to phosphorylation of FAK (focal adhesion kinase), which causes cytoskeletal remodelling, releasing tension on CDH1. This system seemingly resembles a catch-bond model, as found with CTNNA1/VCL and F-actin, and appears to bypass endocytosis and CTNNB1 phosphorylation. It is interesting to note that FAK has more frequently been implicated in WNT/CTNNB1 signalling and EMT in both colorectal and breast cancer [153–155]. Although FAK is more typically associated with cell–matrix interactions at integrin-based contacts, these and other observations suggest that we may have to look beyond this strict subdivision [156]. Note that both of these mechanisms involve CDH1, which would suggest that the adhesive CTNNB1 membrane pool is responsible for the observed translocation.

It should also be pointed out that the models proposed above are not mutually exclusive. It is possible that multiple mechanisms for CTNNB1 translocation from the membrane function alongside each other: the classical endocytosis mechanism leading to turnover of the complete cadherin–catenin complex, which presumably involves the SRC-mediated phosphorylation of Y654, and a tension-mediated mechanism to release CTNNB1, which appears to be Y654-independent. Whether CTNNB1 translocates to the nucleus through changes in tension, phosphorylation or a combination of the two will probably depend on fine-tuned regulatory systems and feedback mechanisms that remain to be elucidated. Although EMT is used as a model system to modulate cell adhesion, this ultimately affects cadherin recycling and tension, which are two biologically concepts relevant in more subtle, physiological situations as well.

## 5. Future perspectives and experimental challenges

Multiple biological and technical challenges remain when it comes to investigating how (patho)-physiological changes in cell adhesion and WNT/CTNNB1 signalling affect the balance between the adhesive and transcriptional functions of CTNNB1, both in healthy tissues and in cancer. Mechanistically, these processes need to be dissected at the molecular level. At the same time, the functional biology and signalling dynamics need to be resolved in living cells and, ultimately, understood in the context of complex tissue morphogenesis and maintenance.

At present, the few studies aimed at tracking the exchange between the different subcellular pools of CTNNB1 have done so in a 2D setting [74,152,157]. Such live-cell imaging approaches hold the promise of visualizing and quantifying dynamic CTNNB1 balance shifts in response to external signals with unprecedented temporal and subcellular resolution. To improve the temporal resolution, future studies will have to consider that both shorter and longer time scales need to be analysed to be able to capture both direct and immediate changes, as well as secondary effects. As an example, during an EMT, initially, the CDH1-containing junctions (and its associated CTNNB1) are removed, but this is followed by the assembly of CDH2-containing membrane contacts that also incorporate CTNNB1. Hypothetically, this could result in a small and transient increase in unbound CTNNB1 that could become available for signalling only temporarily.

The precise dissection of these events, their biological effects and the underlying molecular mechanisms will require the integration of genetic, biochemical and biophysical approaches, which is now within reach thanks to recent technological developments. Genome-editing techniques enable tagging, visualization and quantitative measurements of cellular proteins at endogenous expression levels, allowing us to move away from overexpression studies, which are unlikely to reflect the biological changes during normal physiology [34]. For example, it is typically assumed that AXIN1 levels are limiting under physiological conditions [13,158,159] and it remains to be determined if the biocondensates typically associated with the destruction complex and visible as small cytoplasmic puncta also form in an endogenous context *in vivo*.

Super-resolution microscopy is providing insights into the different CTNNB1-containing protein complexes and may, one day, allow us to discriminate between different functional complexes based on shape, size and the presence of specific interaction partners [23]. Functional imaging studies, such as fluorescence recovery after photobleaching (FRAP) and fluorescence fluctuation spectroscopy, can be used to measure the mobility of different subcellular protein pools [34,74], although, again, these techniques alone do not solve the question of functionality. And unlike earlier *in vitro* biochemical approaches, which were mainly aimed at testing the interaction affinity between two or three individual binding partners, recent advances in proximity labelling and proteomics now allow entire interactomes to be characterized in an unbiased manner, thereby probing the full complexity of the intracellular environment. For instance, a recent study used biotin-dependent proximity labelling of APEX-tagged LRP6 in HEK293T cells to determine changes in the LRP6 co-receptor interactome in response to WNT stimulation [160]. This not only confirmed the presence of known WNT/CTNNB1 signalling components, namely APC, AXIN1 and DVL, in the immediate vicinity of LRP6, but also revealed interactions of LRP6 with the actin cytoskeleton and components of the endocytic machinery.

Ultimately, the question if, and if so, to what extent and under which circumstances crosstalk exists between the different functional pools of CTNNB1 will need to be resolved in 3D space and time, taking tissue- and species-specific differences as well as different developmental time points into account—as the molecular details will undoubtedly vary with the specific cellular context. Some laudable efforts have already been made to study the subcellular distribution of CTNNB1 in intact developing vertebrate animals [161]. Recent developments in gastruloid technology in combination with precision genome editing now also allow exciting opportunities to probe and perturb tissue development in a dish and to study WNT/CTNNB1 in the context of gastrulation movements, not just for mouse but also for human embryonic development [162–165]. Advances in organoid technology hold similar promise for experiments in the context of stem cell turnover and adult tissue maintenance.

In a living cellular organism, cells obviously face completely different circumstances than those that can currently be recapitulated by even the most advanced 3D culture systems. One could argue that with every cell division, which invariably requires dissolution of AJs, order needs to be restored and the distribution of CTNNB1 is reshuffled. This is relevant in all situations where cell proliferation occurs: 2D and 3D cell culture systems, the developing embryo, adult stem cells during tissue maintenance and cancer growth.

*In vivo*, virtually all non-dividing cells are polarized—either in the plane of an epithelial tissue, where they are tightly stacked and organized, or during migration, when they move around in response to a combination of short- and long-distance attractive and repulsive cues. Especially in epithelia, the cell adhesion and transcriptional pools of CTNNB1 may be much more compartmentalized and separated than in a cell culture setting, if only because AJs form laterally and WNT ligands are probably received at the apical or basolateral end [166,167]. In both epithelial and mesenchymal cells, the anterograde and retrograde trafficking of cellular proteins will also occur in a polarized fashion. In short, the 3D spatial organization of cells will greatly affect the WNT signalling process [168] and, by extension, the subcellular distribution of CTNNB1 as well as the extent and kinetics of exchange between the different functional pools.

Finally, it is important to note that while cell adhesion has been modulated in many experimental settings thus far [151,152,157], the modulation of WNT signalling—both the absolute levels of stimulation and the spatial and temporal aspects—has received far less attention and remains greatly understudied. We propose that the precise and systematic perturbation of relevant signalling inputs (i.e. changing the strength of cell adhesion and/or WNT signalling) is critical to resolve in which direction the information flows. Moreover, the membrane, cytoplasmic and nuclear pools of CTNNB1 need to be measured simultaneously and with sufficient spatial and temporal resolution to detect subtle balance shifts of the endogenous protein pool. Ideally, a discrimination should be made between newly synthesized proteins (and their de novo interactions) and the rate of exchange between already existing pools of CTNNB1 (and its associated partners). Finally, relevant and sensitive readouts that allow simultaneous measuring of functional activity (e.g. the presence of active transcriptional complexes) should be included. Connecting phenotypic readouts at the cell and tissue level to the underlying interactions at the gene and protein level will probably continue to pose multiple challenges for the foreseeable future, both when it comes to detection and perturbation of adhesion and WNT signalling and when it comes to selecting the relevant (patho)-physiological model system.

## 6. Conclusion

It remains unclear if CTNNB1 functional pools are kept separated or interact. There is evidence for both sides of the argument, and clearly, the adhesion and transcriptional pools are normally balanced. Even so, few biological states are fixed, let alone binary; cells have a remarkable capacity to adapt to changing circumstances. We propose that future studies should therefore be open to considering a more dynamic model of CTNNB1 regulation that incorporates exchange between the adhesive and transcriptional pools and a continuum of protein states as a result of differential binding and post-translational modification. Such dynamics allow cells to shift and fine tune the balance in cell adhesion and WNT/CTNNB1 signalling as needed.

One exciting possibility therefore remains—which is that the two biological functions of CTNNB1 are actually intrinsically linked and that CTNNB1 functions as a bridge that couples cellular context and gene expression. In the last decade, increasing evidence has shown that mechanical cues from the microenvironment of the cell can be sensed at the plasma membrane and transduced into a signalling response, a process which is called mechanosignalling [169,170]. *In vivo*, cells constantly experience a diverse range of physical forces, resulting from changes in matrix stiffness and composition, physical forces exhibited by neighbouring cells, perturbations on a tissue level, such as blood flow or muscle movement, and intercellular forces coming from actomyosin remodelling within the cells. Cell–cell junctions, including AJs, respond to changes in tension by recruiting different proteins and changing their composition (as reviewed by Angulo-Urarte *et al.* [60]). As CTNNB1 is present at the forefront of mechanosensing as an integral component of AJs, but can simultaneously fulfil a transcriptional role, it would be an ideal candidate to bridge these responses. Indeed, WNT/CTNNB1 signalling has been suggested to be mechanoresponsive itself and linked to the well-known mechanotransducers YAP and TAZ [53,171–177]. These observations support the hypothesis that CTNNB1 might fulfil a core cellular function as a molecular bridge between the cell membrane and the DNA. Such a role would be especially relevant in development and disease, where both the microenvironmental matrix and migratory states are subject to change, which would necessitate altered transcriptional responses to allow cells to adapt and differentiate.

## Data accessibility

This article has no additional data.
